# Varying sensitivity of mountainous streamwater base-flow ${\bf{N}}{{\bf{O}}}_{{\bf{3}}}^{-}$concentrations to N deposition in the northern suburbs of Tokyo

**DOI:** 10.1038/s41598-017-08111-w

**Published:** 2017-08-09

**Authors:** Kazuya Nishina, Mirai Watanabe, Masami K. Koshikawa, Takejiro Takamatsu, Yu Morino, Tatsuya Nagashima, Kunika Soma, Seiji Hayashi

**Affiliations:** 1 National Institute for Environmental Studies, Center for Regional Environmental Research, Tsukuba, 305-8506 Japan; 2 Ibaraki Kasumigaura Environmental Science Center, Tsuchiura, 300-0023 Japan; 3 National Institute for Environmental Studies, Fukushima Branch, Miharu, 963-7700 Japan

## Abstract

Ecosystems of suburban landscapes (i.e., forest, inland water ecosystem) are threatened by high nitrogen (N) loadings derived from urban air pollutants. Forest ecosystems under high chronic N loadings tend to leach more N via streams. In the northern suburbs of Tokyo, N deposition loading on terrestrial ecosystems has increased over the past 30 years. In this region, we investigated nitrate concentrations in 608 independent small forested catchment water samples from northeastern suburbs of Tokyo. The nitrate concentrations varied from 0.07 to 3.31 mg-N L^−1^ in this region. We evaluated the effects of N deposition and catchment properties (e.g., meteorological and topographic factors, vegetation and soil types) on nitrate concentrations. In the random forest model, simulated N deposition rates from an atmospheric chemistry transportation model explained most of the variance of nitrate concentration. To evaluate the effects of afforestation management in the catchment, we followed a model-based recursive partitioning method (MOB). MOB succeeded in data-driven identification of subgroups with varying sensitivities to N deposition rate by vegetation composition in the catchment. According to MOB, the catchment with dominant coniferous coverage that mostly consisted of plantation with old tree age tended to have strong sensitivity of nitrate concentrations to N deposition loading.

## Introduction

Anthropogenic N loading on terrestrial ecosystems have resulted in an perturbation of ecosystem nitrogen (N) cycling, i.e., eutrophication of land water and forest ecosystems^1–3^. Excess reactive N^4^ causes an enhancement of biomass production and biodiversity loss in both terrestrial and aquatic environments^5–10^. Chronic N loading also causes over-nutrition conditions in forest ecosystems^11, 12^. Such forest ecosystems, called “N-saturated forest”, likely have high ${{\rm{NO}}}_{3}^{-}$ concentrations in mountain streamwater and leach more N than the input of reactive N deposition^13^. In the eastern USA, Europe and eastern Asia, some forest ecosystems were reported as having symptoms of N saturation^14–16^. In these regions, several studies have suggested that N deposition explained the spatial gradients of ${{\rm{NO}}}_{3}^{-}$ concentrations from watershed to country scales^17–19^. While Western Europe and North America have stabilized or reduced N deposition in those regions, N deposition has kept high level in East Asian countries, even today^7, 20^.

In the suburbs of Tokyo, several spatial variability studies have reported that air pollutants from the urban area caused higher ${{\rm{NO}}}_{3}^{-}$ concentrations in forested watersheds^21–29^. Throughout the Kanto district (areas surrounding Tokyo), N deposition loadings on terrestrial ecosystems increased in the last decades of the 20th century and have remained high because of high reactive N emissions from the automobile sector^20^. Much attention has been paid to N deposition as a key environmental variable in water quality. However, because it has been difficult to determine multi-site N deposition rates *in situ*, the distance from central Tokyo has been used in studies as an explanatory variable of nitrate concentrations in forested watersheds^23, 26–28, 30^. However, when different scales and directions from the city are included in target area, the distance could not be an universal proxy index of N deposition due to the meteorological factors and topographic factors.

As in prior spatial surveys, N deposition could be an explanatory variable of spatial variation in streamwater ${{\rm{NO}}}_{3}^{-}$. There have been considerable variations in streamwater ${{\rm{NO}}}_{3}^{-}$ concentrations in N-saturated forest, even at the same N deposition rate^13, 17, 19, 31^. Therefore, that rate by itself only weakly explained inter-catchment variations of ${{\rm{NO}}}_{3}^{-}$ concentration. For spatial variations of such concentrations, topographic, vegetation, and geologic factors regulate nitrate leaching from forest watersheds^26, 31, 32^. In addition, these factors may alter N deposition sensitivities of N leaching and ${{\rm{NO}}}_{3}^{-}$ concentration among such watersheds^33^. For management of N deposition impacts on river water quality, it is important to understand how these factors influence N leaching under strong anthropogenic N loading^34^. In particular, from a forest landscape management perspective, it is important to understand which type of forest has strong responses to N deposition^14, 35, 36^.

**Figure 1:**
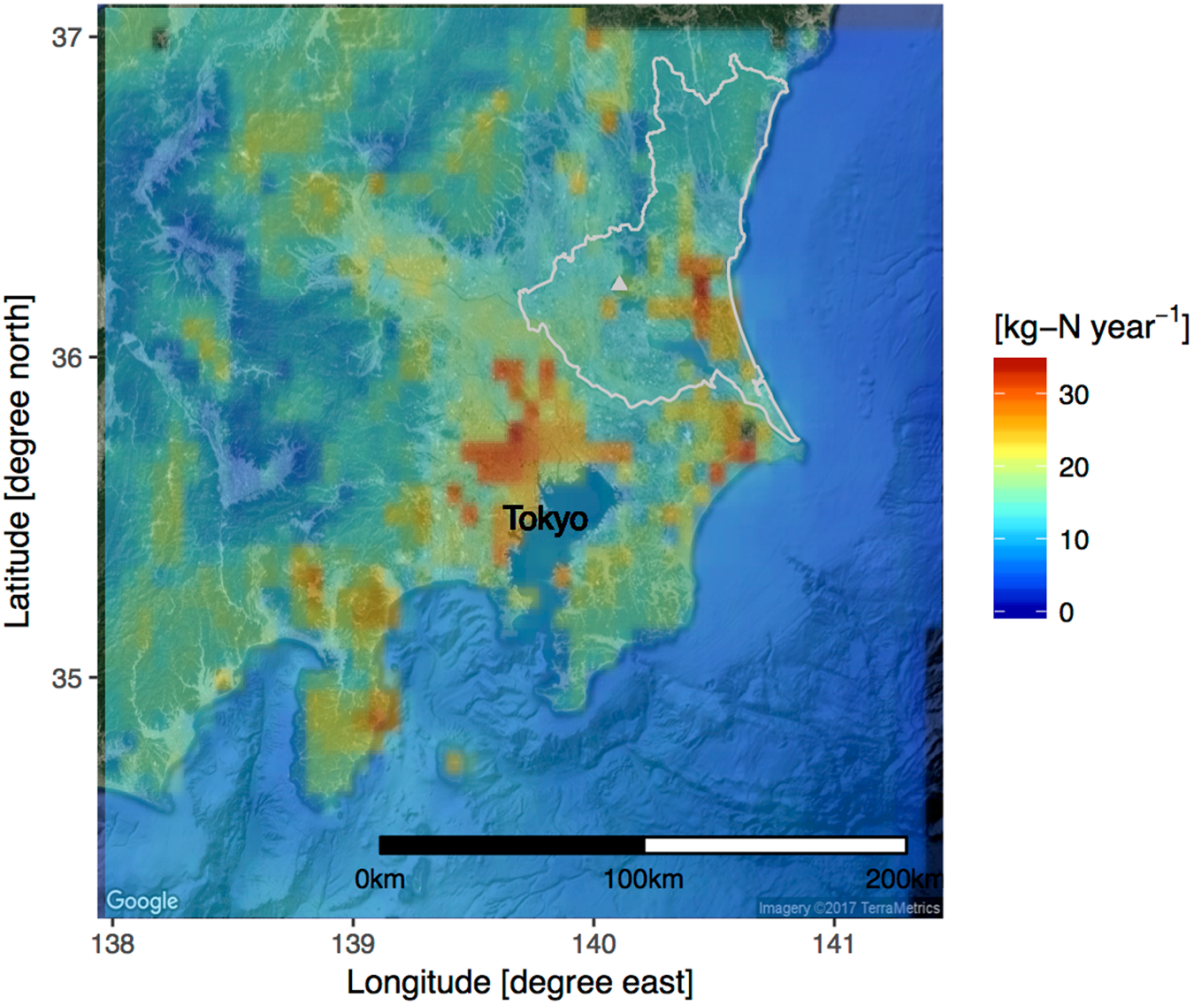
Study area and simulated annual N deposition rate in Kanto region by CMAQ v5.02 model. Solid triangle shows location of Mt. Tsukuba. Background was obtained from ©2016 Google Imagery and ©2016 TerraMetrics. Map was created by ggmap pakage^63^ via Google API.

In the present study, we investigated ${{\rm{NO}}}_{3}^{-}$ concentration in over 600 forest-catchment water samples in the northern suburbs of Tokyo. In the southern part of our study area (around Mount Tsukuba; Fig. 1), the state of N saturation^22^ was observed in the late 1980s^21^. Using intensive spatial sampling with a machine learning technique, we clarified how the N deposition rate and other environmental factors affect base-flow ${{\rm{NO}}}_{3}^{-}$ concentrations in a forested catchment. To obtain the spatial N deposition rate, we used results of atmospheric chemistry transport model simulation^20, 37^ instead of field observation. In addition, based on a large spatial survey, we tried to reveal the effect of afforestation fraction (replacing deciduous forest by conifer species) in catchments on the base-flow ${{\rm{NO}}}_{3}^{-}$ concentration sensitivity to N deposition.

## Methods

### Sampling design

The sampling was done in a forested area (54–675 m a.s.l.) of Mt. Tsukuba and in the northern and southern parts of the Ibaraki mountain system. All study areas were in Ibaraki Prefecture (Fig. 1) and included three major riverine systems (Tone, Naka, and Kuji). These areas are northwest of the Tokyo Metropolis (population about 13 million) and are along the route of frequent passage of polluted air masses from Tokyo^38^. For analysis, we removed samples corrected from the catchment where include different land use type (e.g., cropland) by checking land-use data (described in following section) and satellite images. Finally, We collected water samples from 608 individual forest catchments in the study area during October 2007–August 2010, and put these samples in cold storage before analysis. We avoided sampling water immediately after rain and snowmelt events, i.e., we took samples as least two days after the end of such events.

Prior to the present work, we reported that the base-flow ${{\rm{NO}}}_{3}^{-}$ concentrations during 2010 in 12 catchments adjacent the study area in their research^21^ had comparably high concentrations (about 2 mg-N L^−1^) to those observed in 1986^39^. Moreover, we observed the base-flow ${{\rm{NO}}}_{3}^{-}$ concentration to be stable throughout the year in the southern and northernmost areas of this region (18 and 2 watersheds, respectively; Fig. S1)^39^.

### ${{\rm{NO}}}_{3}^{-}$ and other anion and cation measurements

Electrical conductivity (EC) and pH values were measured in the field using a portable conductivity and pH meter (D-54 and D-55, Horiba, Japan). In all samples, ${{\rm{NO}}}_{3}^{-}$ and other anion contents were measured in the 0.45 μm pore size filtered fraction using ion chromatography (Metrohm, 761 compact IC, Switzerland). Bicarbonate content was determined by acid titration with 0.2 mol L^−1^ H_2_SO_4_. Major cation contents in the filtered fraction were measured by IPC-AES (ICP-750, Thermo Jarrell-Ash, MA).

To verify analytical precision of cations (Na^+^, K^+^, Ca^2+^, Mg^2+^, Sr^2+^) and anions (F^−^, Cl^−^, ${{\rm{HCO}}}_{3}^{-}$, ${{\rm{SO}}}_{4}^{2-}$, and ${{\rm{NO}}}_{3}^{-}$), we calculated the ionic balance error (IBE) on the basis of ions expressed in meq L^−1^. IBE was observed to be within a limit of ±5% for all investigated samples (Table 1, Fig. S2), and therefore we used all samples for statistical analysis. A summary of statistics for water qualities are summarized in Table 1 and a correlation matrix is presented in Fig. S3.

**Table 1:** Statistical summary of water quality (N = 608).

	Mean	St. Dev.	Min	Median	Max
pH	7.3	0.3	5.6	7.3	8.7
EC [μS cm^−1^]	78.9	41	29	67.3	427
NO_3_-N [mg L^−1^]	0.76	0.56	0.07	0.59	3.31
F [mg L^−1^]	0.058	0.087	0.006	0.047	1.66
Cl [mg L^−1^]	4.29	1.83	1.9	3.8	14.3
SO_4_-S [mg L^−1^]	2.33	3.11	0.4	1.4	48.4
HCO_3_ [mg L^−1^]	30.4	17.1	5	26	129
K [mg L^−1^]	0.73	0.35	0.11	0.64	2.32
Na [mg L^−1^]	5.89	2.77	2.12	5.42	41.22
Ca [mg L^−1^]	7.11	5.72	1.66	5.54	47.75
Mg [mg L^−1^]	2.00	1.52	0.38	1.51	18.92
Si [mg L^−1^]	9.75	2.58	3.87	9.61	18.99
Sr [mg L^−1^]	0.04	0.02	0.01	0.04	0.23
Ionic balance [%]	−1.51	1.27	−4.83	−1.59	3.65

### Geographic and spatial characteristics of sampling catchment

To acquire geographic information of the catchment, we used a digital elevation model (DEM) with 10-m resolution, which is published by the Geographical Survey Institute of Japan. For statistical analysis, elevations were taken at sampling points. To delineate catchment boundaries for each sampling point, we used a geographic information system (ArcGIS) and its Hydrology and ArcHydro tools. After specification of the catchment, mean topographic variables of the catchment (area, slope and aspect) were calculated. For aspect, we used the aspect southness which values was the cosine of the azimuth *θ* (−cos(*θ*)). This index range from −1 to 1, which indicates of how south or north a site faces. This conversion can avoid the difficulties in interpretation of azimuth because 0 and 360 signify the same aspect.

We used climate factors for each catchment from 1-km mesh data of National Land Numerical Information^40^. We used annual mean air temperature, annual precipitation, and annual radiation, which are representative of the 1981–2010 average. We referred to the land-use map created for biodiversity assessment based on the fifth national vegetation map constructed by the Japanese Ministry of the Environment (1994–1998)^41, 42^. This map has 10-m resolution. From the associated dataset, we used vegetation type information (e.g., coniferous, deciduous) for forest area and calculated the fraction of each type. In the study region, almost all coniferous forests are plantation and mainly consist of *Cryptomeria japonica* and *Chamaecyparis obtusa*. Almost all deciduous forest in the region is secondary forest, with typical dominant canopy species *Fagus japonica*, *Quercus serrata*, *Kalopanax pictus*, *Acer mono f*. *marmoratum*, *Quercus crispula Blume*, and *Fagus crenata* (particularly at high elevation).

We used simulated annual N deposition (including both dry and wet depositions) for 2010 in the region from results of Community Multi-scale Air Quality (CMAQ) model version 5.0.2 with the sixth-generation aerosol module^43^, which was validated and calibrated using national acid rain census data in refs 20 and 37. Anthropogenic emission data from Japan and East Asia were from the Japan AutoOil Program^44, 45^ and Regional Emission Inventory in Asia version 2.1^46^, respectively. Data from other sources are summarized in ref. 37. We established three simulation domains. Domain 1 covered East Asia with horizontal resolution 60 km, Domain 2 covered Japan with horizontal resolution 15 km, and Domain 3 covered the Kanto region with horizontal resolution 5 km. Results in Domain 3 were analyzed.

Abbreviations of each variable and statistical summary for the sampled catchment properties from GIS and CMAQ outputs are shown in Table 2.

**Table 2:** Statistical summary of sampled catchment (N = 608).

Variable	Abb.*	Mean	S.D.	Min	Median	Max
Longitude [°]		140.384	0.178	140.047	140.350	140.733
Latitude [°]		36.599	0.207	36.156	36.654	36.926
Area [ha]	Area	51.69	57.61	0.34	35.45	644.85
Elevation [m]	Elev	350	159	82	315	817
Slope [°]	Slope	24.5	4.0	13.8	24.1	37.6
Aspect southness	Aspect	0.84	0.22	−0.75	0.92	1.00
Precipitation [mm year^−1^]	Prep	1511	166	1267	1439	2018
Temperature [°C]	Temp	11.5	1.1	8.1	11.7	13.6
Radiation [W m^−2^]	Rad	12.8	0.15	12.4	12.8	13.4
N deposition [kg-N ha^−1^ year^−1^]	Ndep	13.6	2.7	8.0	13.1	22.4
Broad-leaved coverage [%]	BL	20.4	25.9	0.0	7.7	100.0
^†^Evergreen conifer coverage [%]	ND	70.5	29.6	0.0	79.4	100.0
Evergreen broad-leaved coverage [%]	EG	0.21	3.51	0	0	72.36
^†^Deciduous conifer coverage [%]	DC	0.01	0.24	0	0	5.83
Brown Forest Soil [%]	BFsoil	91.52	22.3	0.0	100.0	100.0
Andosol [%]	ADsoil	6.8	19.9	0.0	0.0	100.0
Grey Lowland Soil [%]	GLsoil	0.57	4.25	0	0	52.74
Brown Lowland Soil [%]	BLsoil	0.65	7.09	0	0	100
Grey Soil [%]	Gsoil	0.5	5.9	0.0	0.0	100.0

*Indicates abbreviations in the results of random forest model (Fig. 3). S.D. indicates standard deviation. Forest coverages with ^†^indicate afforested forests (i.e., plantation).

### Statistical analysis

To predict catchment-to-catchment variations in ${{\rm{NO}}}_{3}^{-}$ concentrations using environmental variables (summarized in Table 2) and evaluate their relative importance, we used a random forest algorithm^47^ using the randomForest package^48^ in the R environment^49^. We used a total of 400 data (randomly selected) as a training dataset and the rest (N = 209) as validation data (i.e., out of bag sample). For growing trees in a random forest, the number of trees were set to 500 and that of predictors tested at each node was set to 8; the latter value was determined by searching for the optimal value with respect to validation data. To evaluate model fit, we calculated a coefficient of determination R^2^. Variable of importance in RF regression were calculated from the percentage increase in out-of-bag mean square error when features were removed one-by-one from the model^48^. Through analysis of the relative importance of individual variables, useful information was obtained concerning the relative importance of all variables and their capability to forecast ${{\rm{NO}}}_{3}^{-}$ concentration. For RF regression, we used 17 explanatory variables including N deposition, climate factors (Temp, Prep, Radiation), topographic factors (area of catchment, elevation, slope, aspect southness), coverage of each vegetation types (e.g., broad-leaved, evergreen-conifer) (see Table 2 in detail).

Second, to investigate the effect of afforestation fraction (replacing deciduous forest by conifer species) of the catchments by sensitivities to N deposition of ${{\rm{NO}}}_{3}^{-}$ concentration, we used model-based recursive partitioning methods (MOB), known as hybrid trees^50^. This approach is based on recursive partitioning methods for generalized linear models, i.e., splitting data into subset groups of observations with different linear model parameters *θ*. The basic concept of this model is to assume a series of linear model “${ {\mathcal M} }_{ {\mathcal B} }$” for a number of subset datasets “${\mathcal B}$” if the model of whole and partial datasets (${\rm{ {\mathcal M} }}$) is not sufficiently stable in fitting^50^. If a global model for all “n” observations does not fit well and additional covariates ${{\bf{Z}}}_{1},\ldots ,{{\bf{Z}}}_{l}$ are available, it might be possible to partition *b* observations with respect to these variables by the tree-like function $f({{\bf{Z}}}_{1},\ldots ,{{\bf{Z}}}_{l})$. Then, we determine a local model fit for each cell of the partition. We set a single linear model with ${{\rm{NO}}}_{3}^{-}$ concentration as an independent variable Y and annual N deposition rate as an explanatory variable “X”. Coniferous (EN) and deciduous (DB) coverages were set as partitioning variables “**Z**” in the MOB analysis. For fitting in this model, we used the “mob” function in R package “party”^50^. To avoid overfitting, pre-pruning was implemented using the Akaike Information Criterion (AIC); the minimum sample size in each subset which was set to N = 50. Using this model, we evaluated potential interactions through a model-based recursive partitioning algorithm, testing candidate main effect terms (N deposition in this study). Also, we fitted a simple linear model to the global (whole) dataset as a reference. All statistical treatments were done in the R environment^49^.

## Results and Discussion

**Figure 2:**
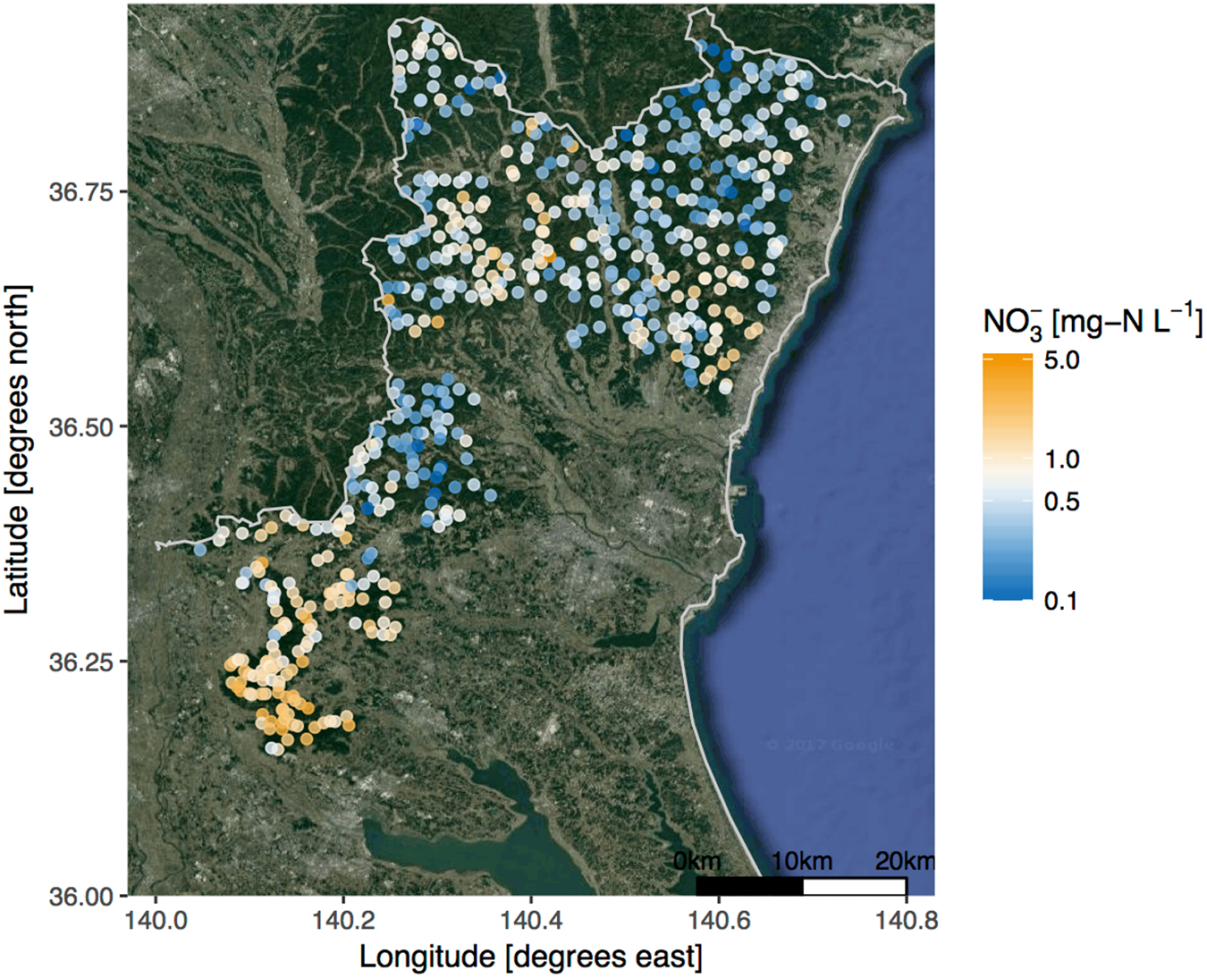
${{\rm{NO}}}_{3}^{-}$ concentrations of forest watersheds in Ibaraki. Background was obtained from ©2016 Google Imagery and ©2016 TerraMetrics. Map was drawn by ggmap package^63^ via Google API.

In the sampling area, simulated annual N deposition from the CMAQ model varied from 8.0 to 22.4 kg-N ha^−1^ (Fig. 1). This range and spatial gradients of the simulated N depositions were well agreed with the observations especially in the mountainous area in previous studies^28, 38, 51^ (See comparison in Fig. S4; R^2^ = 0.855 and RMSE = 1.12 kg-N ha^−1^). Also, this range is comparable with N deposition for N-saturated forest ecosystems reported in European, North American, and East Asian countries^17, 18, 52^. In all stream samples, ${{\rm{NO}}}_{3}^{-}$ concentration ranged from 0.07 to 3.31 mg-N L^−1^, and its median was 0.56 mg-N L^−1^ (median absolute deviation; ±0.40 mg-N L^−1^) (Fig. 2). The concentration range is generally comparable with that from a Japan-wide stream monitoring campaign (N = 1278)^25^. In the present study, about 25% of samples were greater than 1 mg-N L^−1^ as ${{\rm{NO}}}_{3}^{-}$, which was a larger distribution ratio than that in Japan-wide monitoring^25^. Such high N concentration samples in the study region are comparable with values in European forest^17, 18^ and the same Kanto-region forest catchment reported to have N-saturated forests^25–27^. However, contrary to the European forests, pH in 99% of samples were >6.5, suggesting streams without acidification despite relatively high acid deposition loadings. Ohte *et al*.^53^ stated that streams with high ${{\rm{NO}}}_{3}^{-}$ but not low pH are commonly observed in Japanese N-saturated forest. Their research suggests that this characteristic of Japanese streamwater is attributable to substantial base cations in young volcanic ash material^54^. The high ${{\rm{HCO}}}_{3}^{-}$ concentrations (30.4 ± 17.1 mg L^−^) in our samples (Table 1) support this hypothesis.

**Figure 3:**
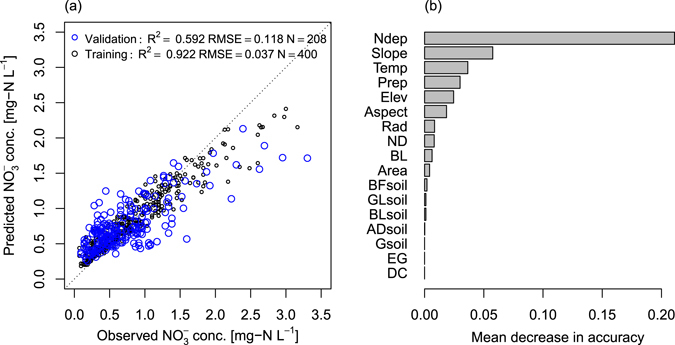
Predicted versus observed values of ${{\rm{NO}}}_{3}^{-}$ conc. for both training and validation data by RF model (**a**) and, variable of importance to the ${{\rm{NO}}}_{3}^{-}$ conc. variation in RF model (**b**). Variable of importance are shown as mean decrease in accuracy.

The ${{\rm{NO}}}_{3}^{-}$ concentrations showed spatial gradients, i.e., high in the south and low in the northern mountainous area (Fig. 2). The random forest model demonstrated that our predictions of base-flow water chemistry at independent validation sites were sufficiently precise and accurate (R^2^ = 0.592 for validation dataset) to reveal spatial ${{\rm{NO}}}_{3}^{-}$ concentration variation (Fig. 3(a)). The RF model showed that N deposition rate was the most important variable in ${{\rm{NO}}}_{3}^{-}$ concentration variation (Fig. 3(b)), suggesting that the spatial trends substantially originated from spatial N deposition differences between regions (Fig. 3). However, the N deposition rate in RF model was not an actual observation but CMAQ simulation output. CMAQ model provides complete spatial coverage over the study area, which N deposition is reflected in the local emission inventory, micro-climate conditions (differences in precipitation, wind direction by topography), and land-use, by simulating formation and transportation of air pollutants^20^. In this study area, we found good agreement with CMAQ outputs and observations in annual N deposition, though in small sample size (Fig. S4). It is important advantage that the ability to estimate N deposition in locations where monitoring data are not existing. On the other hands, major issues in the simulated N deposition are prediction ability (needs more validation) and coarse resolution (5 km × 5 km = 2500 ha) relative to the small forest catchment scale (mean area in this study was 52 ha). Therefore, the simulated N deposition could not capture adjusted inter-catchment differences of actual N deposition. However, we believed that using the other environmental factors could compensate this inconsistency of scale in the RF model. For example, slope direction (southern index in the present study) might affect N deposition of the catchment as controlled by airflow (Tokyo is south of our sites). In fact, it is difficult to measure accurate N deposition rates at catchment scale *in situ*, owing to heterogeneity of the topography and forest crown. Hence, the simulated N deposition could be the best approach at present and is useful as an explanatory variable in the regional scale evaluation. In RF model, we also found that the slope, annual temperature and precipitations of the catchment had positive relationship with ${{\rm{NO}}}_{3}^{-}$ concentrations.

**Figure 4:**
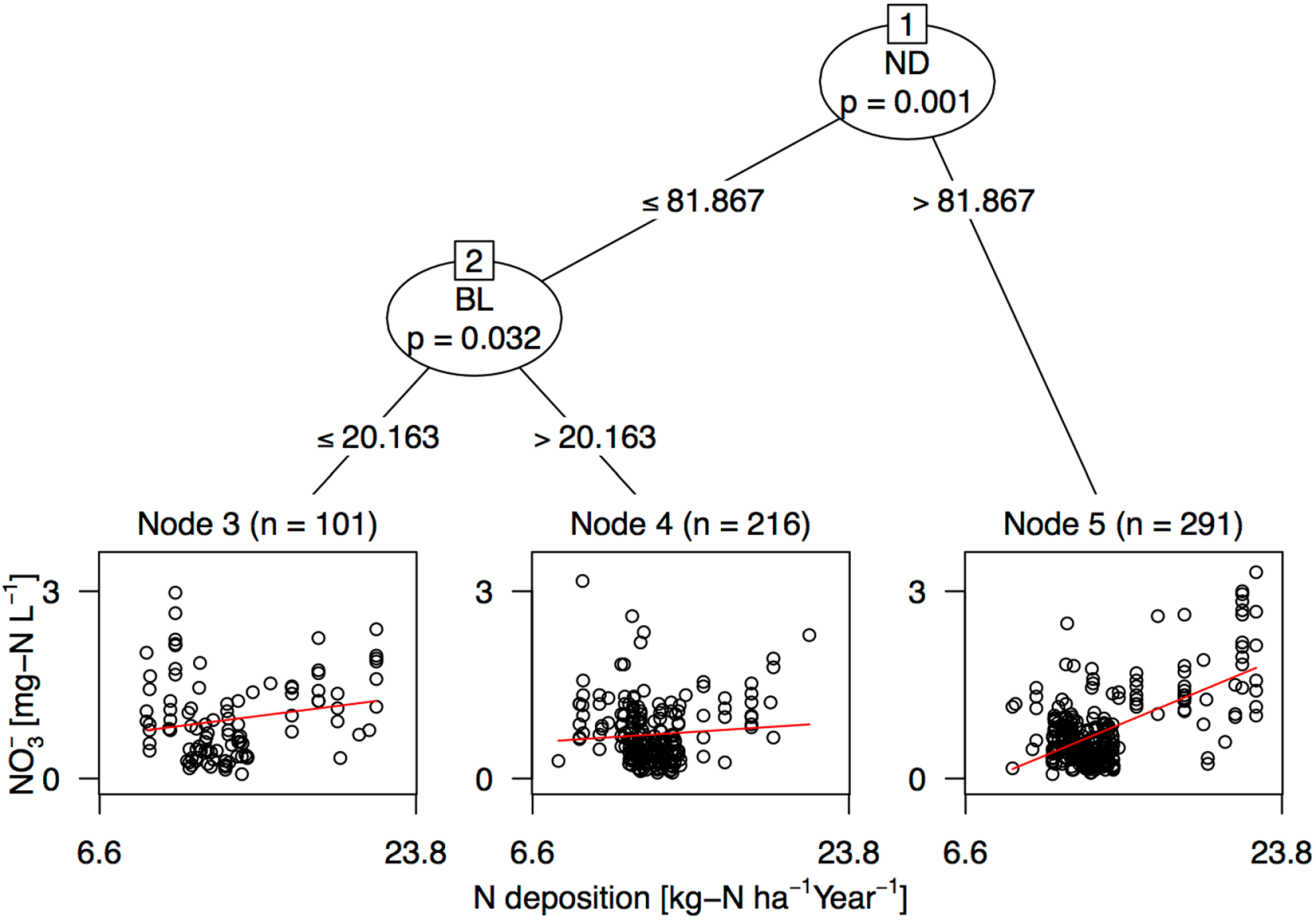
Recursively partitioned linear regression model of ${{\rm{NO}}}_{3}^{-}$ concentrations explained by N deposition. Detailed information for fitted model is given in Table 3. ND and BL are Needle and Broaf Leaves trees, respectively. The units for values under the node are % of coverage for each vegetation type.

Because the N deposition was estimated as a main effect term in the RF model (Fig. 3(b)), we used MOB to identify how the catchment vegetation (afforestation) distribution interacted with the sensitivity to N deposition of spatial ${{\rm{NO}}}_{3}^{-}$ concentration trends. MOB methods statistically differentiated three subset groups using two factors (ND and BL coverages), whose groups had different coefficients of intercept and slope vs. the N deposition rate (Fig. 4 and Table 3). MOB analysis considerably improved the AIC compared to a linear model of the whole dataset (Table 3). The highest node was firstly differentiated by ND (coniferous coverage of the catchment), which threshold was 82%. The second node (node 2) was clustered by BL, whether the BL is $\leqq$20.2% or >20.2%. We found the highest regression slope in node 5, which group of the catchments was dominated by ND (Fig. 4 and Table 3). These results suggests the catchment with dominant coniferous coverage (i.e., afforested area) had strong sensitivity to N deposition rate in the base-flow ${{\rm{NO}}}_{3}^{-}$ concentration (Fig. 4). That is, such catchments in the study area had strong sensitivity to N deposition of N leaching (at least to the base-flow ${{\rm{NO}}}_{3}^{-}$ concentrations). In the study area, 92% (58%) of artificial forests (including both Japanese cedar and cypress) are greater than 30 (50) years old^55^, partially because of delays in forest management caused by a forestry decline in Japan. Such age compositions in the artificial forest, which are prominent throughout the country, might have produced the strong N-deposition sensitivity in the catchment with high needleleaf coverage. Early works^13, 30, 34^ pointed out that growing forests leach less N that mature ones under the same N deposition. Tree age was an important factor for the uptake and maintenance of N in aboveground biomass^14, 34^. This has also been observed in Japanese artificial forest^30, 56^. For example, in the chronosequence of uniformly aged Japanese cedar stands, N uptake by tree biomass decreased, especially for ages greater than 16 years^57^. This is because of the reduction of tree growth with age. In addition, unmanaged old-age forest are likely to have very low biomass in their understory^58^. Another possible mechanism for the varying sensitivity caused by vegetation differences is the ability of deposition absorption by the forest crown, owing to variations in LAI and crown roughness^59^. With the same extent of LAI and canopy heights, coniferous species with needle leaves have higher capture rates than broadleaf trees during dry and fog deposition^60–62^. Thus, the canopy structural differences may be attributed to sensitivity varying with the slope of ${{\rm{NO}}}_{3}^{-}$ concentration vs. simulated N deposition among catchments with different vegetation. Recently, it was reported that N leaching differences between coniferous plantations and evergreen broadleaf forest increased during storm flow conditions in the southwestern part of Japan^56^. Therefore, to evaluate the sensitivity differences of N leaching among forest types under strong N deposition, more careful sampling of base-flow conditions are needed to clarify the impacts of that deposition in Japanese suburban forests.

**Table 3:** Statistical summary of linear models in MOB (Fig. 4) and whole dataset.

Model/Node	Coefficient	Estimate	S.D.	*t* value	*p* value
MOB model (N = 608, AIC = 905)					
Node 3 (N = 101)	Intercept	0.522	0.287	1.82	0.072
	N deposition	0.031	0.020	1.52	0.133
Node 4 (N = 216)	Intercept	0.472	0.210	2.26	0.025
	N deposition	0.017	0.016	1.05	0.296
Node 5 (N = 291)	Intercept	−0.924	0.133	−6.96	<0.01
	N deposition	0.117	0.009	12.57	<0.01
Linear regression for whole dataset (N = 608, AIC = 2700)					
	Intercept	−0.246	0.110	−2.25	0.025
	N deposition	0.072	0.008	9.20	<0.01

## Electronic supplementary material

Supplemental figures
