# Supplementary material for: Varying sensitivity of mountainous streamwater base-flow NO3−concentrations to N deposition in the northern suburbs of Tokyo
Source: Sci Rep. 2017 Aug 9;7:7701. doi: 10.1038/s41598-017-08111-w (PMC5550466; doi:10.1038/s41598-017-08111-w)
Supplement: Supplementary file 1 — Supplemental figures [file 41598_2017_8111_MOESM1_ESM.pdf]

Supplemental materials (4 figures)

Varying sensitivity of mountainous streamwater base-flow NO_3^- concentrations to N deposition in the northern suburbs of Tokyo

Kazuya Nishina, Mirai Watanabe, Masami K. Koshikawa, Takejiro Takamatsu
Yu Morino, Tatsuya Nagashima, Kunika Soma, and Seiji Hayashi

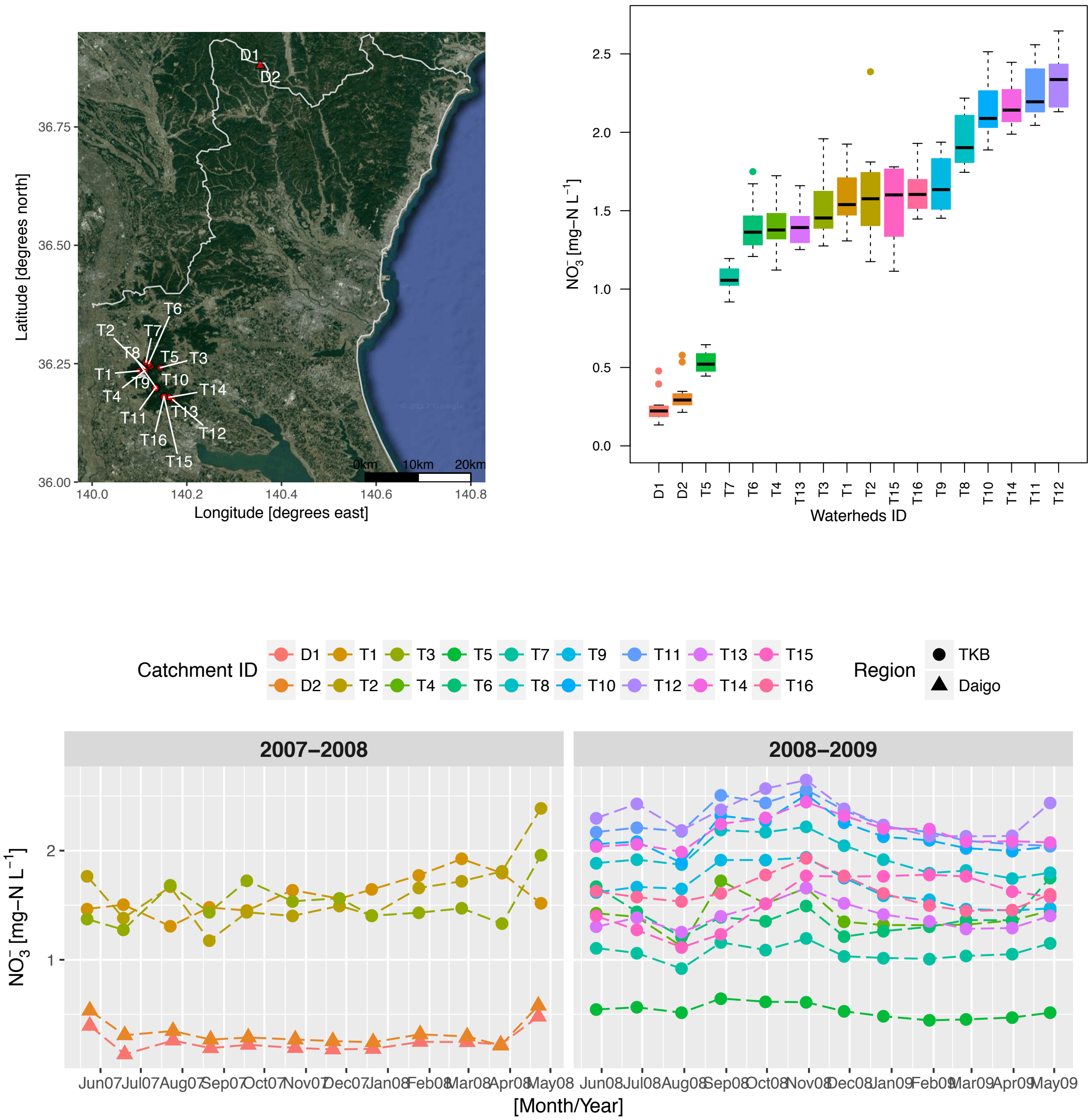

Figure S1. Preliminary surveys for base-flow NO_3^- concentrations (at an approximately monthly interval) throughout a year at 18 watersheds (N = 12 in each). Location of observations (left) summary of NO_3^- concentrations at each watershed as boxplot (right), and time-series NO_3^- concentrations at each watershed (bottom). The samplings were conducted in 2 seasons (2007–2008 for D1, D2, and T1–3; and 2008–2009 for T4–16). These watersheds were consisted of conifers (Japanese Cedar and Japanese cypress) and not overlapped with the main sampling for spatial surveys of this study. Background in the map was obtained from ©2016 Google Imagery and ©2016 TerraMetrics. Map was created by ggmap package via Google API.

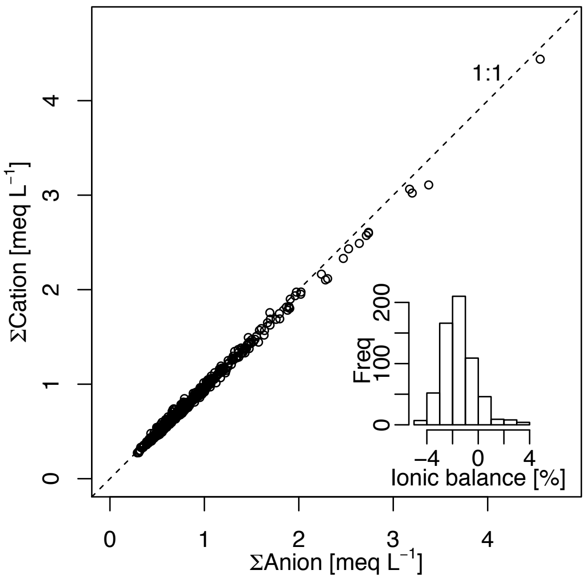

Figure S2. Ionic equivalent for cations (Na^+, K^+, Ca^{2+}, Mg^{2+}, Sr^{2+}) and anions (F^-, Cl^-, HCO_3^-, SO_4^{2-}, and NO_3^-) and ionic balance error (in histogram plot).

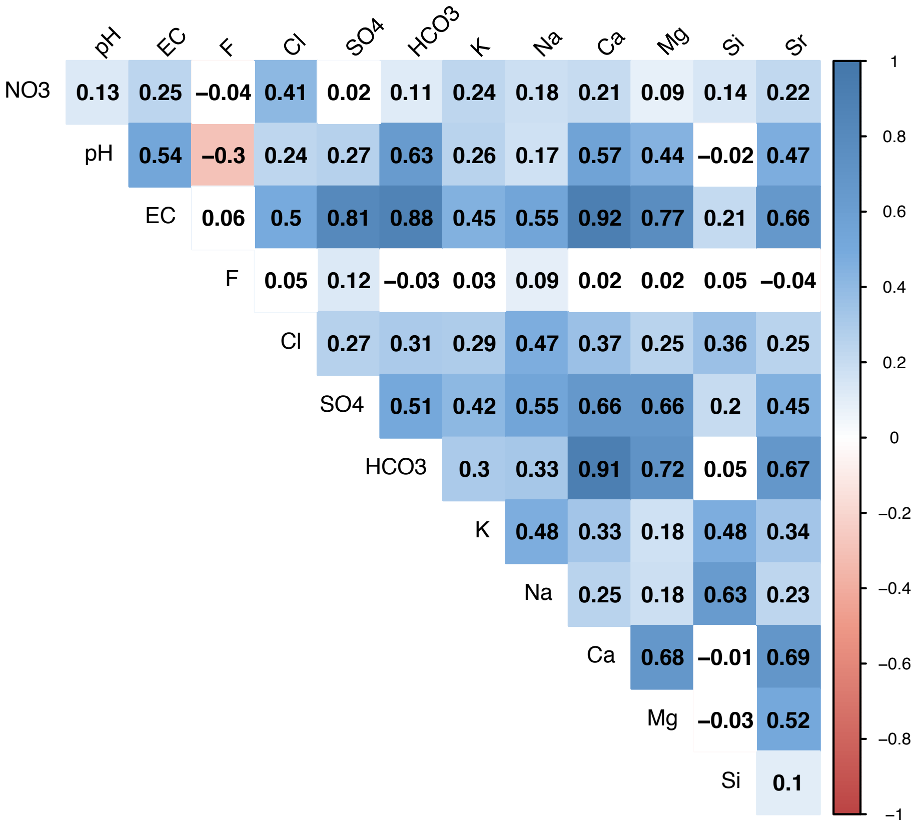

Figure S3. Pearson's correlation between major ions and water quality indices. Color gradients indicate statistical significance from correlation coefficients ($p < 0.05$).

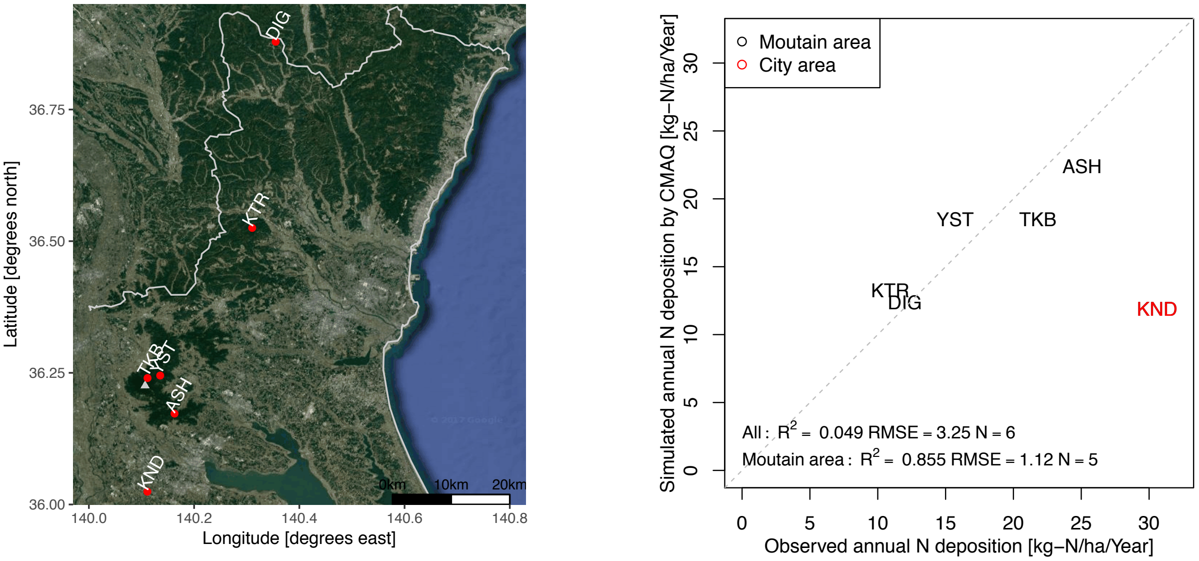

Figure S4. Comparison between simulated N deposition by CMAQ model and observed N deposition as throughfall input in previous studies in this region. Location of observations (left) and comparison (right). The values in KND (red pine) and YST (deciduous) were reported in Oura et al. (2001). The values in DIG (Japanese Cedar) and TKB (Japanese Cedar) were reported in Takamatsu et al. (2010). The values in YST (Japanese Cedar) and KTR (Japanese Cedar) were reported in Yoshinaga et al. (2012). Background in the map was obtained from ©2016 Google Imagery and ©2016 TerraMetrics. Map was created by ggmap pakage[62] via Google API.
